# Serial laboratory biomarkers are associated with ICU outcomes in patients hospitalized with COVID-19

**DOI:** 10.1371/journal.pone.0293842

**Published:** 2023-11-07

**Authors:** Xinan Wang, Emma White, Francesca Giacona, Amita Khurana, Yi Li, David C. Christiani, Jehan W. Alladina

**Affiliations:** 1 Department of Environmental Health, Harvard T. H. Chan School of Public Health, Harvard University, Boston, MA, United States of America; 2 Division of Pulmonary and Critical Care Medicine, Department of Medicine, Massachusetts General Hospital, Boston, MA, United States of America; 3 Department of Biostatistics, School of Public Health, University of Michigan, Ann Arbor, MI, United States of America; Universitas Pelita Harapan, INDONESIA

## Abstract

**Background:**

Clinical utility of routinely measured serial biomarkers in predicting escalation of inpatient care intensity and mortality among hospitalized patients with COVID-19 remains unknown.

**Methods:**

This retrospective cohort study included patients with COVID-19 who admitted to the Massachusetts General Hospital between March and June 2020 and January to March 2021. White blood cell (WBC) count, platelet count, C-reactive protein (CRP), and D-dimer values were measured on days 1, 3, and 7 of admission. Clinical outcomes include 30- and 60-day morality, ICU transfer, and overall survival (OS) over a follow-up period of 90 days. The association between serial biomarkers and outcomes were assessed using multivariable logistic regression and Cox proportional hazards models.

**Measurements and main results:**

Of the 456 patients hospitalized with COVID-19, 199 (43.6%) were ICU, 179 (39.3%) were medical floor, and 78 (17.1%) were initially admitted to the medical floor and then transferred to the ICU. In adjusted analyses, each unit increase in the slope of CRP was associated with a 42% higher odds of ICU transfer after controlling for the initial admission level (OR = 1.42, 95% CI: 1.25–1.65, P < 0.001). Including serial change in CRP levels from initial level on admission achieved the greatest predictive accuracy for ICU transfer (AUC = 0.72, 95% CI: 0.64–0.79).

**Conclusions:**

Serial change in CRP levels from admission is associated with escalations of inpatient care intensity and mortality among hospitalized patients with COVID-19.

## Introduction

Coronavirus disease 2019 (COVID-19) is a respiratory infection that can present with a broad spectrum of clinical manifestations ranging in severity [1]. While 40–50% of COVID-19 cases are asymptomatic, a significant proportion of patients may develop rapidly progressive respiratory failure and other complications that require intensive inpatient care, such as hospitalization, supplemental oxygen therapy, and mechanical ventilation [2–4]. Patients who present to the hospital have heterogeneous disease courses and clinical outcomes that may include respiratory failure, multi-organ dysfunction, and death in the most severe cases. It is imperative for clinicians to have access to readily obtainable and clinically meaningful biomarkers to prognosticate the clinical course of disease and thereby effectively triage and optimize the therapeutic options for patients.

Given the global pandemic, there have been a number of studies evaluating the use of biomarkers for disease prognosis among hospitalized patients with COVID-19 [5–8]. Previous studies have shown that low lymphocyte, platelet counts and albumin levels, and elevated C reactive protein (CRP), white blood cell (WBC) count, blood urea nitrogen, interleukin-6 (IL-6), blood urea nitrogen, creatine kinase, procalcitonin, D-dimer, lactate dehydrogenase, alanine aminotransferase, aspartate aminotransferase and creatinine levels are all associated with poor COVID-19 outcomes [9–20]. The Sequential Organ Failure Assessment (SOFA) score is associated with worse clinical outcomes among intensive care unit (ICU) patients as well as patients with COVID-19 pneumonia requiring mechanical ventilation [21, 22].

However, there have been few studies on serial changes in laboratory biomarkers for prognosis and clinical outcomes among hospitalized patients with COVID-19. Understanding the variation and profile of obtainable clinical biomarkers since admission and how they are related to COVID-19 outcomes may facilitate the development of risk-stratified approaches and timely clinical decision-making that would benefit hospitalized patients [9]. To fill this important knowledge gap, we investigated the associations of baseline and serial changes in WBC count, platelet count, CRP, and D-dimer from whole blood samples that were consecutively measured on days 1, 3, and 7 since admission with a variety of clinical outcomes, including 30- and 60-day morality, ICU transfer, and overall survival (OS) among the 456 hospitalized patients with COVID-19 who were admitted to Massachusetts General Hospital (MGH) between March and June 2020 (surge 1) and January to March 2021 (surge 2).

## Materials and methods

### Patient recruitment and sample collection

We studied adult ICU patients with SARS-CoV-2 infection and acute hypoxemic respiratory failure (AHRF) and non-ICU patients with mild hypoxemia managed with 2–6 L/min supplemental oxygen who were consecutively admitted to MGH between March and June 2020 (surge 1) and January to March 2021 (surge 2) retrospectively. Informed consent was waived, and the study was approved by the institutional review board of MGH (protocol number 2015P001650). We collected baseline demographics, clinical characteristics, and outcomes from the electronic medical record. We assessed comorbidities, including body mass index (BMI), previous pulmonary disease, chronic obstructive pulmonary disease (COPD), asthma, and interstitial lung disease (ILD). We calculated modified sequential organ failure assessment (mSOFA) score on day 1 for ICU patients [23–25]. WBC count (1000/mm^3^), platelet count (1000/mm^3^), CRP (mg/L), and D-dimer (ng/ml) were consecutively measured on days 1, 3, and 7 since admission by the MGH clinical core laboratory. We report all available data without imputation, and the statistical analyses were conducted between August 2022 and February 2023

### Clinical outcomes

30- and 60-day mortality were calculated, respectively, as the probabilities of death within 30 and 60 days after admission. Every patient was followed for up to 90 days after admission and OS was defined as from the date of hospital admission to death or the date of last contact, whichever happened first. ICU transfer was documented for those transferred to the ICU from medical floor after admission.

### Statistical analysis

Descriptive statistics summarized the baseline demographic and clinical data. Categorical variables were reported with counts and percentages and continuous variables were reported with median and range. Kruskal-Wallis tests and chi-square tests were used for continuous and categorical variables, respectively. Logistic regression was used to evaluate the association with binary outcomes (30- and 60-day mortality and ICU transfer). Association analyses with ICU transfer were conducted in patients who were initially admitted to the medical floor. Event-time distributions were estimated using Kaplan-Meier methodology. Log-rank tests were used to test the differences in event-time distributions and Cox proportional hazards models were fitted to estimate hazard ratios (HR). Potential confounders of age, gender, smoking status, ethnicity and COVID surge were adjusted for in the multivariable analyses. The slope of serial changes in each biomarker for each patient was obtained to assess its time-varying effects with the clinical outcomes.

To address whether serial changes in biomarkers from admission provide additional predictive values on clinical outcomes, area under the curve (AUC) and C-index were calculated and compared between 1) baseline models with only biomarker level on day 1, and 2) models incorporating serial changes and levels on day 1 in combination with baseline covariates. The receiver operating characteristic curve (ROC) and area under the curve (AUC) were generated and calculated based on 5-fold cross-validation for logistic regression, while the C-index was calculated for the Cox proportional hazard model in survival analysis. 95% confidence intervals (CIs) for AUC and C-index were obtained by creating 300 bootstrap samples and taking the 2.5^th^ and 97.5^th^ percentiles of the re-estimates. All reported *P*-values are two-sided and confidence intervals are at the 95% level, with significance pre-defined to be at < 0.05.

## Results

### Demographic and clinical characteristics

**Fig 1:**
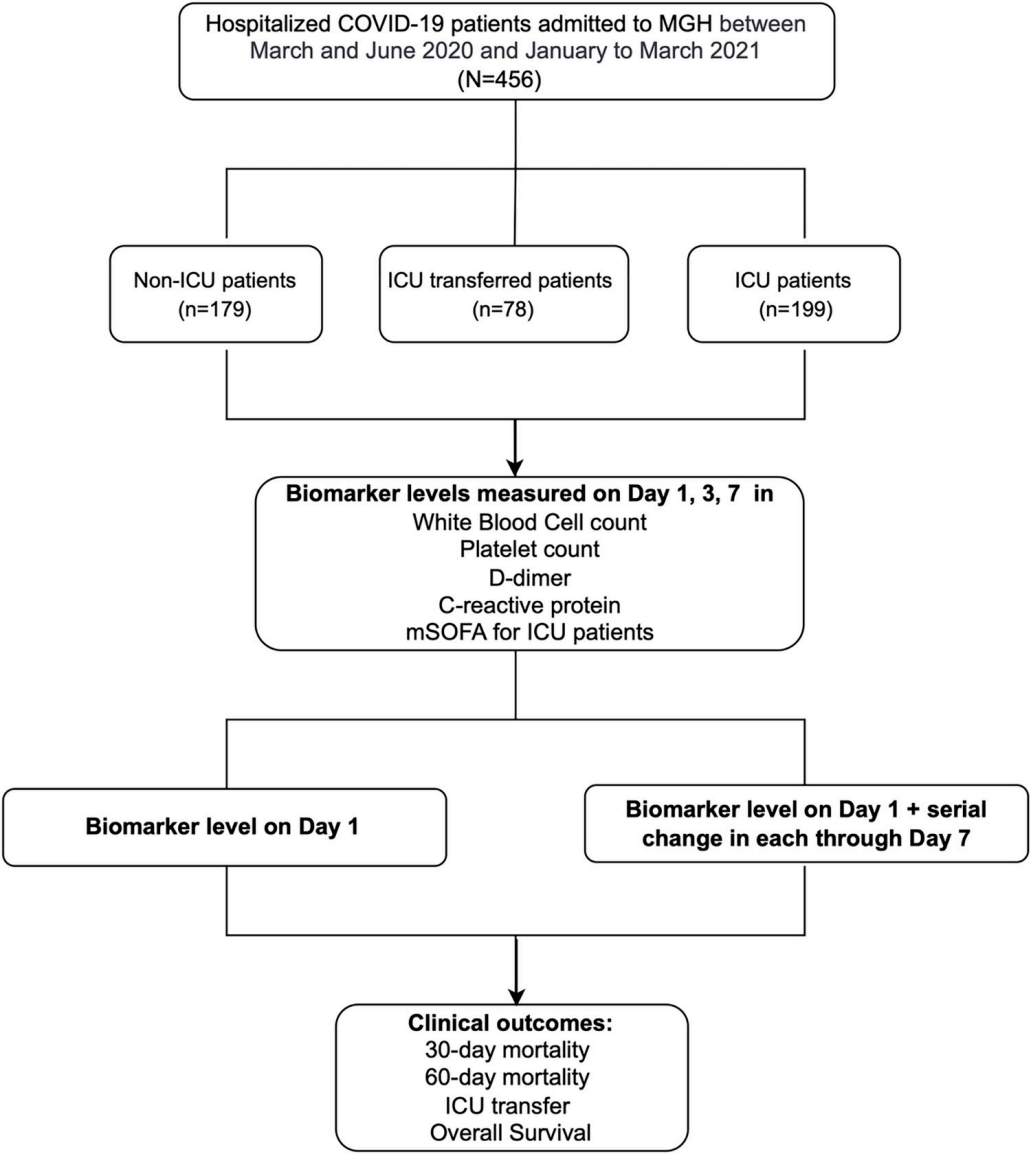
Study overview.

Of the 456 hospitalized COVID patients, 199 (43.6%) were admitted to ICU, 179 (39.3%) were admitted to the medical floor (non-ICU), and 78 (17.1%) were transferred to ICU after admission to the medical floor (**Fig 1**). Patient demographics, clinical characteristics, and clinical outcomes are summarized by COVID severity in **S1 Table**. A total of 182 (39.9%) patients were admitted during the second surge of COVID-19. The median age at diagnosis was 63 years (range, 51–73 yr), 282 (61.8%) patients were male, and 154 (33.8%) patients were Hispanic/Latino. 184 (40.4%) patients were former or current smokers, and 129 (28.3%) patients had preexisting pulmonary diseases, including 65 (14.3%) with asthma, 53 (11.6%) with COPD, and 7 (1.5%) with ILD.

The study was conducted in 456 hospitalized COVID-19 patients admitted to MGH between March and June 2020 (surge 1) and January to March 2021 (surge 2). Among them, 199 (43.6%) were admitted to ICU, 179 (39.3%) were non-ICU patients and 78 (17.1%) were transferred to ICU after admission, Laboratory biomarkers in white blood cell (WBC) count, platelet count, D-dimer, and C-reactive protein (CRP) were consecutively measured on day 1, 3, and 7 since admission for all while modified sequential organ failure assessment (mSOFA) at ICU admission was measured for ICU patients. We assessed the prognostic effects of each biomarker level on day 1 as well as the serial change in each controlling for their corresponding values on day 1, along with baseline covariates on 30-day, 60-day mortality, ICU transfer and overall survival with a follow-up period of 90 days.

**Fig 2:**
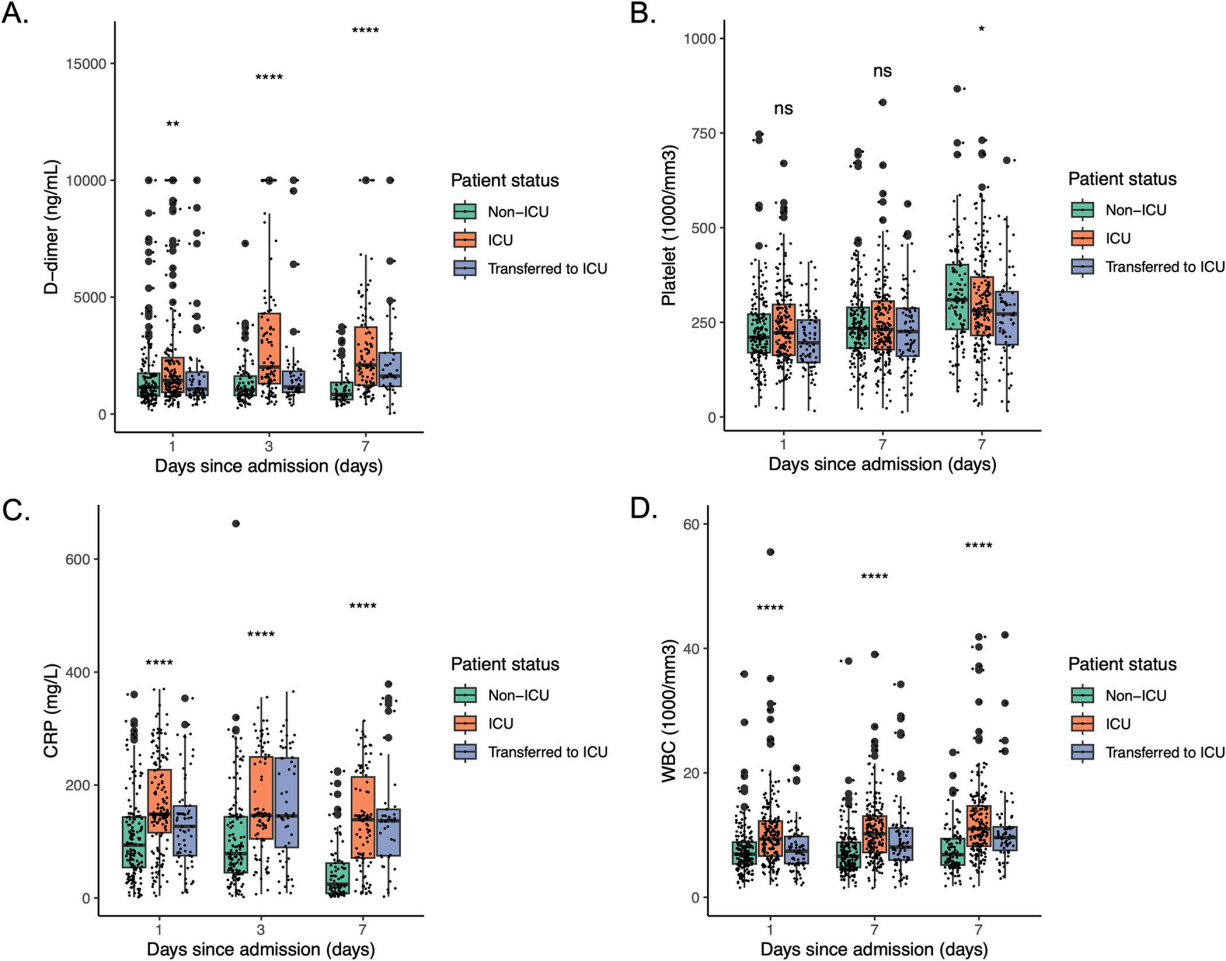
Serial biomarker levels on day 1, 3 and 7 since admission by COVID severity. (A) D-dimer (ng/ml) level on day 1, 3 and 7 since admission for COVID-19 patients by severity. (B) Platelet count (1000/mm^3^) level on day 1, 3 and 7 since admission for COVID-19 patients by severity. (C) C-reactive protein (mg/L) level on day 1, 3 and 7 since admission for COVID-19 patients by severity. (D) White blood cell (WBC) count (1000/mm^3^) level on day 1, 3 and 7 since admission for COVID-19 patients by severity.

Laboratory values of WBC count (1000/mm^3^), platelet count (1000/mm^3^), CRP (mg/L) and D-dimer (ng/ml) on day 1 of admission for all patients and mSOFA score for ICU patients were summarized in **S2 Table**. A significantly higher WBC count, CRP and D-dimer values on day 1 of admission were observed for ICU patients (median WBC (mWBC): 9.4 (1000/mm^3^) range: 2.0–168 (1000/mm^3^); median CRP (mCRP): 148 (mg/L) range: 3.7–370 (mg/L); median D-dimer (mD-dimer): 1440 (ng/ml) range: 302–10000 (ng/ml), respectively) compared to non-ICU patients (mWBC: 6.9 (1000/mm^3^) range: 1.5–35.9 (1000/mm^3^); mCRP: 93.6 (mg/L) range: 1.4–360 (mg/L); mD-dimer: 1140 (ng/ml) range: 163–10000 (ng/ml), respectively). Serial biomarker levels on day 1, 3, 7 of admission stratified by COVID severity are shown in **Fig 2**. The median mSOFA was 7 (range, 1–15) for ICU patients. ICU patients had significantly higher WBC counts and CRP and D-dimer levels on day 3 and 7 of admission, compared to non-ICU patients, as described in **S2 Table**.

### Association between biomarker levels on admission and clinical outcomes

Among ICU patients, mSOFA on admission is associated with increased 30-day mortality (OR = 1.39, 95% CI: 1.21–1.62, P < 0.001) and 60-day mortality (OR = 1.42, 95% CI: 1.24–1.66, P < 0.001), as well as increased overall mortality (HR = 1.30, 95% CI: 1.18–1.44, P < 0.001) after adjusting for age, gender, smoking status, ethnicity, and COVID surge (**S3 Table**). Higher CRP level on day 1 of admission is associated with increased 30- and 60-day mortality as well as increased odds of ICU transfer (for every 10 mg/L increase in CRP: 30-day mortality OR = 1.04, 95% CI: 1.01–1.08, P = 0.02; 60-day mortality OR = 1.05, 95% CI: 1.01–1.09, P = 0.01; ICU transfer OR = 1.04, 95% CI: 1.01–1.09, P = 0.02, respectively). Every 1000 ug/mL increase in D-dimer on day 1 of admission is significantly associated with a 14% increase in 30-day mortality (OR = 1.14, 95% CI: 1.02–1.28, P = 0.04) and a trend towards an increase in 60-day mortality (OR = 1.12, 95% CI: 0.99–1.25, P = 0.07). Furthermore, CRP, D-dimer, platelet count, and WBC count on day 1 of admission are all significantly associated with OS (every 10 mg/L increase in CRP: HR = 1.04, 95% CI: 1.02–1.07, P = 0.002; every 1000 ug/mL increase in D-dimer: HR = 1.10, 95% CI: 1.01–1.20, P = 0.03; every 1000/mm3 increase in platelet count: HR = 0.997, 95% CI: 0.995–1.000, P = 0.03; every 1000/mm3 increase in WBC count: HR = 1.02, 95% CI: 1.01–1.03, P = 0.001, respectively) (**Table 1**).

**Table 1:** Association between biomarkers on day 1 of admission and clinical outcomes.

*n = 381*	30-day mortality			60-day mortality			ICU transfer (n = 229)			Overall Survival		
	OR	95% CI	P	OR	95% CI	P	OR	95% CI	P	HR	95% CI	P
**Every 10mg/L increase in CRP**	1.04	1.01–1.08	0.02	1.05	1.01 – 1.09	0.01	1.04	1.01 – 1.09	0.02	1.04	1.02–1.07	0.002
**Age**	1.09	1.06 – 1.12	<0.001	1.08	1.06 – 1.11	<0.001	1.01	0.99 – 1.04	0.24	1.08	1.06 – 1.10	<0.001
**Female vs. Male**	0.58	0.31 – 1.07	0.09	0.56	0.31 – 1.02	0.06	0.79	0.43 – 1.45	0.45	0.72	0.44 – 1.17	0.18
**Ever smoker vs. never smoker**	0.68	0.36 – 1.27	0.23	0.74	0.40 – 1.34	0.32	0.49	0.24 – 0.97	0.04	0.75	0.45 – 1.23	0.25
**Surge 2 vs. 1**	1.04	0.56 – 1.90	0.89	1.16	0.64 – 2.08	0.62	0.65	0.34 – 1.22	0.19	1.10	0.69 – 1.76	0.68
**Hispanic vs. Non-Hispanic**	1.01	0.48 – 2.08	0.98	0.94	0.46 – 1.90	0.87	0.87	0.42 – 1.77	0.71	0.87	0.48 – 1.58	0.65
***n = 377***	**30-day mortality**			**60-day mortality**			**ICU transfer (n = 225)**			**Overall Survival**		
	**OR**	**95% CI**	**P**	**OR**	**95% CI**	**P**	**OR**	**95% CI**	**P**	**HR**	**95% CI**	**P**
**Every 1000 ug/mL increase in D-dimer**	1.14	1.01–1.28	0.04	1.12	0.99–1.25	0.07	1.07	0.89–1.27	0.45	1.10	1.01–1.20	0.03
**Age**	1.09	1.06 – 1.12	<0.001	1.08	1.05 – 1.11	<0.001	1.01	0.99 – 1.03	0.50	1.07	1.05 – 1.10	<0.001
**Female vs. Male**	0.56	0.29 – 1.05	0.08	0.56	0.30 – 1.03	0.07	0.94	0.51 – 1.72	0.85	0.67	0.40 – 1.12	0.13
**Ever smoker vs. never smoker**	0.68	0.35 – 1.31	0.26	0.74	0.39 – 1.37	0.34	0.63	0.31 – 1.23	0.18	0.73	0.44 – 1.23	0.24
**Surge 2 vs. 1**	0.82	0.43 – 1.55	0.55	1.00	0.54 – 1.82	1.00	0.59	0.30 – 1.13	0.12	0.98	0.60 – 1.60	0.94
**Hispanic vs. Non-Hispanic**	0.74	0.33 – 1.61	0.47	0.80	0.37 – 1.66	0.56	1.01	0.50 – 2.02	0.97	0.80	0.42 – 1.52	0.49
***n = 429***	**30-day mortality**			**60-day mortality**			**ICU transfer (n = 248)**			**Overall Survival**		
	**OR**	**95% CI**	**P**	**OR**	**95% CI**	**P**	**OR**	**95% CI**	**P**	**HR**	**95% CI**	**P**
**Platelet count (1000/mm3)**	0.997	0.993–0.999	0.02	0.996	0.993–0.999	0.01	0.997	0.994–1.000	0.09	0.997	0.995–1.00	0.03
**Age**	1.07	1.05 – 1.09	<0.001	1.07	1.04 – 1.09	<0.001	1.00	0.98 – 1.03	0.65	1.06	1.04 – 1.08	<0.001
**Female vs. Male**	0.54	0.30 – 0.93	0.03	0.56	0.32 – 0.95	0.03	0.82	0.46 – 1.44	0.49	0.72	0.47 – 1.12	0.14
**Ever smoker vs. never smoker**	0.82	0.47 – 1.43	0.49	0.86	0.50 – 1.47	0.59	0.59	0.31 – 1.11	0.11	0.81	0.52 – 1.25	0.35
**Surge 2 vs. 1**	1.17	0.69 – 1.98	0.57	1.39	0.83 – 2.31	0.21	0.74	0.41 – 1.32	0.31	1.27	0.84 – 1.91	0.26
**Hispanic vs. Non-Hispanic**	1.04	0.53 – 1.98	0.91	1.07	0.56 – 2.00	0.83	1.01	0.51 – 1.97	0.98	0.98	0.58 – 1.67	0.94
***n = 431***	**30-day mortality**			**60-day mortality**			**ICU transfer (n = 249)**			**Overall Survival**		
	**OR**	**95% CI**	**P**	**OR**	**95% CI**	**P**	**OR**	**95% CI**	**P**	**HR**	**95% CI**	**P**
**WBC count (1000/mm3)**	1.03	1.00 – 1.08	0.09	1.03	1.00 – 1.07	0.11	1.03	0.98 – 1.08	0.28	1.02	1.01 – 1.03	0.001
**Age**	1.07	1.05 – 1.09	<0.001	1.07	1.04 – 1.09	<0.001	1.01	0.99 – 1.03	0.54	1.06	1.04 – 1.08	<0.001
**Female vs. Male**	0.51	0.29 – 0.89	0.02	0.52	0.30 – 0.88	0.02	0.77	0.43 – 1.36	0.37	0.69	0.45 – 1.07	0.10
**Ever smoker vs. never smoker**	0.77	0.44 – 1.34	0.37	0.84	0.49 – 1.42	0.52	0.61	0.32 – 1.14	0.13	0.83	0.54 – 1.28	0.40
**Surge 2 vs. 1**	1.04	0.61 – 1.76	0.89	1.28	0.77 – 2.13	0.34	0.73	0.40 – 1.31	0.30	1.19	0.79 – 1.79	0.42
**Hispanic vs. Non-Hispanic**	1.00	0.51 – 1.93	0.99	1.00	0.53 – 1.87	1.00	1.03	0.52 – 2.00	0.93	0.97	0.56 – 1.66	0.90

### Prognostic associations of serial changes in biomarker levels after admission

We also investigated the association between biomarker levels on Day 7 of admission after adjusting for levels on Day 1 and other baseline covariates, and similar findings were summarized in **S4 Table**. We next evaluated the prognostic associations of serial changes in biomarker levels on clinical outcomes by including in the regression models the slope for each patient along with the corresponding biomarker level on day 1 and baseline covariates (**Table 2**). Increasing CRP levels were associated with worse prognosis and clinical outcomes. Each unit increase in the slope of CRP was associated with a 12% increase in 30- and 60-day mortality (30-day mortality OR = 1.12, 95% CI: 1.03–1.21, P = 0.008; 60-day mortality OR = 1.12, 95% CI: 1.04–1.22, P = 0.005), a 42% increase in ICU transfer (OR = 1.42, 95% CI: 1.25–1.65, P < 0.001) and reduced OS (HR = 1.07, 95% CI: 1.01–1.14, P = 0.02). Similarly, each unit increase in the slope of WBC was associated with increased 30-day mortality (OR = 3.85, 95% CI: 1.90–8.05, P < 0.001), 60-day mortality (OR = 2.91, 95% CI: 1.47–5.87, P < 0.001), higher risk of ICU transfer (OR = 9.43, 95% CI: 2.92–34.65, P < 0.001) and increased overall mortality (HR = 1.99, 95% CI: 1.24–3.20, P = 0.004). In contrast, increasing platelet counts were associated with improved clinical outcomes; with each unit increase in the slope was associated with a 6% decrease in 30-day (OR = 0.94, 95% CI: 0.92–0.97, P < 0.001) and 60-day mortality (OR = 0.94, 95% CI: 0.91–0.96, P < 0.001), a 4% decrease in odds of ICU transfer (OR = 0.96, 95% CI: 0.94–0.99, P = 0.01) and less overall mortality (HR = 0.96, 95% CI: 0.94–0.97, P < 0.001). An increase in the slope of D-dimer was only modestly associated with an increased risk of ICU transfer after controlling for level on day 1 of admission and baseline covariates (OR = 1.01, 95% CI: 1.00–1.01, P = 0.01).

**Table 2:** Prognostic effects of serial changes in biomarkers since admission.

*n = 381*	30-day mortality			60-day mortality			ICU transfer			Overall Survival		
	OR	95% CI	P	OR	95% CI	P	OR	95% CI	P	HR	95% CI	P
**CRP (mg/L) at Day 1**	1.01	1.00 – 1.01	0.001	1.01	1.00 – 1.01	<0.001	1.02	1.01 – 1.02	<0.001	1.01	1.00 – 1.01	<0.001
**Slope of CRP**	1.12	1.03 – 1.21	0.008	1.12	1.04 – 1.22	0.005	1.42	1.25 – 1.65	<0.001	1.07	1.01 – 1.14	0.02
**Age**	1.09	1.06 – 1.12	<0.001	1.08	1.06 – 1.11	<0.001	1.01	0.98 – 1.03	0.63	1.08	1.05 – 1.10	<0.001
**Female vs. Male**	0.58	0.30 – 1.07	0.09	0.56	0.30 – 1.01	0.06	0.71	0.36 – 1.38	0.32	0.71	0.43 – 1.16	0.17
**Ever smoker vs. never smoker**	0.74	0.39 – 1.39	0.36	0.80	0.43 – 1.48	0.48	0.50	0.23 – 1.04	0.07	0.80	0.49 – 1.32	0.39
**Surge 2 vs. 1**	1.31	0.69 – 2.49	0.40	1.48	0.79 – 2.76	0.22	0.91	0.44 – 1.82	0.78	1.35	0.81 – 2.24	0.25
**Hispanic vs. Non-Hispanic**	1.04	0.49 – 2.17	0.91	0.97	0.47 – 1.98	0.94	0.96	0.43 – 2.10	0.91	0.90	0.50 – 1.62	0.72
*n = 377*	**30-day mortality**			**60-day mortality**			**ICU transfer**			**Overall Survival**		
	**OR**	**95% CI**	**P**	**OR**	**95% CI**	**P**	**OR**	**95% CI**	**P**	**HR**	**95% CI**	**P**
**D-dimer (ug/mL) at Day 1**	1.00	1.00 – 1.00	0.36	1.00	1.00 – 1.00	0.18	1.00	1.00 – 1.00	0.02	1.00	1.00 – 1.00	0.31
**Slope of D-dimer**	1.00	1.00 – 1.00	0.82	1.00	1.00 – 1.00	0.68	1.01	1.00 – 1.01	0.01	1.00	1.00 – 1.00	0.94
**Age**	1.09	1.06 – 1.12	<0.001	1.08	1.05 – 1.11	<0.001	1.01	0.99 – 1.03	0.55	1.07	1.05 – 1.10	<0.001
**Female vs. Male**	0.55	0.29 – 1.05	0.07	0.57	0.30 – 1.05	0.07	0.99	0.53 – 1.84	0.98	0.67	0.40 – 1.12	0.13
**Ever smoker vs. never smoker**	0.69	0.35 – 1.32	0.26	0.73	0.39 – 1.36	0.33	0.60	0.29 – 1.18	0.15	0.73	0.44 – 1.24	0.25
**Surge 2 vs. 1**	0.82	0.43 – 1.54	0.54	1.01	0.55 – 1.84	0.97	0.62	0.31 – 1.21	0.17	0.98	0.60 – 1.60	0.93
**Hispanic vs. Non-Hispanic**	0.74	0.33 – 1.61	0.47	0.80	0.37 – 1.66	0.56	1.07	0.53 – 2.15	0.86	0.80	0.42 – 1.52	0.49
*n = 429*	**30-day mortality**			**60-day mortality**			**ICU transfer**			**Overall Survival**		
	**OR**	**95% CI**	**P**	**OR**	**95% CI**	**P**	**OR**	**95% CI**	**P**	**HR**	**95% CI**	**P**
**Platelet count (1000/mm3) at Day 1**	0.99	0.99 – 1.00	0.001	0.99	0.99 – 1.00	<0.001	1.00	0.99 – 1.00	0.04	1.00	0.99 – 1.00	0.001
**Slope of platelet**	0.94	0.92 – 0.97	<0.001	0.94	0.91 – 0.96	<0.001	0.96	0.94 – 0.99	0.01	0.96	0.94 – 0.97	<0.001
**Age**	1.06	1.04 – 1.08	<0.001	1.06	1.04 – 1.08	<0.001	1.00	0.98 – 1.02	0.83	1.05	1.03 – 1.07	<0.001
**Female vs. Male**	0.55	0.31 – 0.97	0.04	0.57	0.32 – 0.99	0.05	0.77	0.43 – 1.37	0.38	0.78	0.50 – 1.21	0.26
**Ever smoker vs. never smoker**	0.92	0.52 – 1.63	0.79	0.98	0.56 – 1.72	0.95	0.62	0.32 – 1.17	0.14	0.88	0.57 – 1.37	0.58
**Surge 2 vs. 1**	1.04	0.60 – 1.79	0.88	1.23	0.72 – 2.09	0.44	0.72	0.39 – 1.29	0.27	1.20	0.80 – 1.80	0.38
**Hispanic vs. Non-Hispanic**	1.34	0.67 – 2.63	0.40	1.40	0.72 – 2.71	0.31	1.29	0.64 – 2.59	0.47	1.14	0.67 – 1.93	0.63
*n = 431*	**30-day mortality**			**60-day mortality**			**ICU transfer**			**Overall Survival**		
	**OR**	**95% CI**	**P**	**OR**	**95% CI**	**P**	**OR**	**95% CI**	**P**	**HR**	**95% CI**	**P**
**WBC count (1000/mm3) at Day 1**	1.05	1.01 – 1.10	0.01	1.05	1.01 – 1.09	0.02	1.06	1.01 – 1.13	0.03	1.01	0.99 – 1.03	0.25
**Slope of WBC**	3.85	1.90 – 8.05	<0.001	2.91	1.47 – 5.87	0.002	9.43	2.92 – 34.65	<0.001	1.99	1.24 – 3.20	0.004
**Age**	1.07	1.05 – 1.10	<0.001	1.07	1.04 – 1.09	<0.001	1.00	0.98 – 1.02	0.84	1.06	1.04 – 1.08	<0.001
**Female vs. Male**	0.51	0.28 – 0.89	0.02	0.52	0.30 – 0.89	0.02	0.90	0.49 – 1.63	0.72	0.68	0.44 – 1.05	0.09
**Ever smoker vs. never smoker**	0.78	0.44 – 1.37	0.39	0.85	0.49 – 1.45	0.55	0.66	0.34 – 1.27	0.22	0.76	0.49 – 1.18	0.22
**Surge 2 vs. 1**	0.81	0.46 – 1.41	0.46	1.07	0.63 – 1.81	0.81	0.45	0.23 – 0.87	0.02	1.07	0.70 – 1.63	0.75
**Hispanic vs. Non-Hispanic**	0.89	0.45 – 1.74	0.74	0.91	0.47 – 1.72	0.77	0.93	0.46 – 1.86	0.85	0.87	0.51 – 1.50	0.63

### ROC analyses

**Fig 3:**
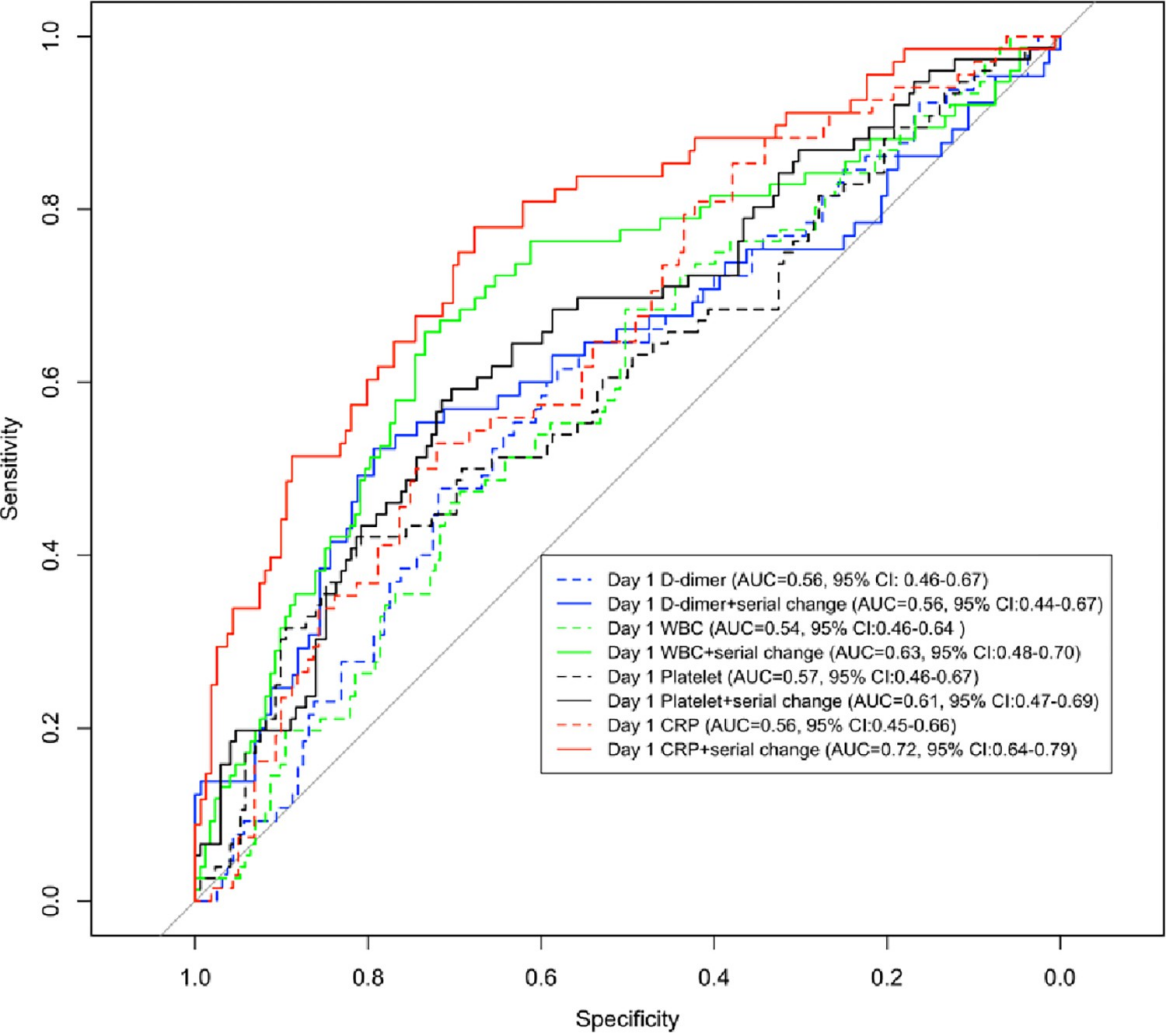
ROC analyses for ICU transfer.

Incorporating serial changes in biomarkers provided incremental predictive values in all clinical outcomes, with the largest improvement in predicting the need for ICU transfer (**Fig 3, S5 Table**). Including the slope of CRP change since admission achieved the highest AUC and largest improvement in predicting ICU transfer (AUC = 0.72, 95% CI: 0.64–0.79) compared to the baseline model (AUC = 0.56, 95% CI: 0.45–0.66), suggesting potential clinical utility in triaging severe patients urgently in need of closer monitoring and more intensive care support.

Area under the curve (AUC) for ICU transfer was calculated in each biomarker: D-dimer, WBC count, platelet count and CRP, for both models in incorporating 1) only biomarker level on day 1, and 2) serial changes over time and corresponding level on day 1, in combination with baseline covariates of age, gender, smoking status, ethnicity and COVID surge.

## Discussion

While the majority of patients with COVID-19 have mild symptoms, those admitted to the hospital may experience rapid disease progression, requiring ventilatory support and ICU admission despite the increasing prevalence of vaccine administration and adjunctive therapies. Biomarkers that predict disease progression and clinical outcomes may facilitate the triage of patients with worsening disease in need of close monitoring and intensive care support, enabling efficient allocation of limited and precious healthcare resources [3]. To our knowledge, our results demonstrated for the first time that, in addition to the laboratory biomarkers measured on day 1 of admission, increases in CRP levels and WBC count, and decreases in platelet count are associated with escalation of inpatient care intensity and poor clinical outcomes among hospitalized patients with COVID-19.

The SOFA score, a mortality prediction score based on the degree of dysfunction of six organ systems, has been well adopted in COVID triage policies [26]. Consistent with prior studies, increased mSOFA score on day 1 of admission was associated with increased mortality among ICU patients [16, 21, 22]. Our study shows that elevated D-dimer, CRP, WBC count and decreased platelet count on day 1 of admission were also associated with higher risks of mortality and ICU transfer. Prior studies show that higher D-dimer level on day 1 of admission predicts higher in-hospital mortality [13, 27]. CRP, an acute inflammatory biomarker, has been widely used for assessing the severity of inflammatory and infectious conditions. Our study confirmed other small cohort studies and meta-analyses that conjectured that an elevated CRP level is associated with higher mortality and provided novel findings regarding ICU transfer [9, 28–30].

However, very few studies investigated the prognostic and predictive effects of serial biomarkers in hospitalized COVID-19 patients. Serial measurements of time-varying cardiac biomarkers, including high-sensitivity cardiac troponin (hsTnI) and NT-proBNP, predict a higher mortality rate and show an enhanced association with OS compared to the baseline level on day 1 of admission among hospitalized patients [31, 32]. Our study is innovative in characterizing the predictive value of serial measurement in D-dimer, CRP, WBC count, and platelet count on mortality, ICU transfer and OS among hospitalized COVID-19 patients. Incorporating serial changes in these biomarkers improves the model performance in all clinical outcomes, with the highest and largest increase in predicting ICU transfer using CRP. The higher mortality in patients with an increasing trend in WBC count can be due to treatment effects, generalized systemic inflammation and secondary bacterial infection, and the biological mechanisms merit further investigation. Taken together, our results suggested that consecutive measurements of these biomarkers beyond baseline concentrations are associated with COVID-19 progression course and clinical outcomes in hospitalized patients.

Our study has several limitations. First, this is a single-center retrospective study based on a sample of hospitalized COVID-19 patients admitted from March and June 2020 and January to March 2021, implying that the estimates may not be transferable with less virulent strains and in the vaccine era. Second, while there are missing data for CRP and D-dimer, it doesn’t change our main conclusion that serial change in CRP predicts ICU escalation; indeed, this only further strengthens the association as potential bias introduced could only be towards the null. Third, detailed immunosuppressive treatment information is not available in this work. However, the surge indicator may serve as a surrogate for steroid usage, and it was adjusted in the analyses. Fourth, as our study population was predominantly composed of White, non-Hispanic patients, our findings should be further tested in a larger, more diverse population in a prospective study for improved generalizability. While we do not have detailed microbiology data on the patients included in this study, it is certainly possible that some of the findings may be attributable to secondary bacterial infection. Our study was limited by its retrospective design, and some data were unavailable from the electronic medical records. Additionally, there were inconsistencies in the missingness of biomarkers at different time points, potentially reducing the accuracy of our estimates and model performances.

In conclusion, we have demonstrated that elevated mSOFA score, D-dimer, CRP, WBC count and decreased platelet count on day 1 of admission were associated with higher 30- and 60-day mortality, higher ICU transfer and more overall mortality among hospitalized COVID-19 patients. Serial CRP, WBC count, and platelet count are readily available in clinical practice and provide additional predictive value for disease progression and clinical outcomes, supporting the use of serial laboratory biomarkers in the triage and monitoring of hospitalized patients with COVID-19.

## Supporting information

S1 File (PDF)

S1 Table Baseline demographic and clinical characteristics by COVID severity. (DOCX)

S2 Table Biomarker levels by COVID severity. (DOCX)

S3 Table Association between mSOFA on day 1 and clinical outcomes among ICU patients. (DOCX)

S4 Table Association between biomarkers on day 7 and clinical outcomes adjusting for levels on day 1 since admission. (DOCX)

S5 Table ROC analyses for 30-day, 60-day mortality, ICU transfer and OS. (DOCX)

## Abbreviations

COVID-19: Coronavirus disease 2019
ICU: Intensive Care Unit
WBC: White blood cell
CRP: C-reactive protein
mSOFA: Modified sequential organ failure
AUC: Area Under the Curve
ROC: Receiver Operating characteristic Curve
OR: Odds Ratio
HR: Hazard Ratio
CI: Confidence Interval
OS: Overall Survival
