# Supplementary material for: Serial laboratory biomarkers are associated with ICU outcomes in patients hospitalized with COVID-19
Source: PLoS One. 2023 Nov 7;18(11):e0293842. doi: 10.1371/journal.pone.0293842 (PMC10629639; doi:10.1371/journal.pone.0293842)
Supplement: S1 File — (PDF) [file pone.0293842.s001.pdf]

Mass General Brigham IRB
Mass General Brigham
399 Revolution Drive, Suite 710
Somerville, MA 02145
Tel: 857-282-1900
Fax: 857-282-5693

Notification of IRB Review

Protocol #: 2015P001650

Date:	June 15, 2022
To:	Alladina, Jehan<br>MGH<br>Mass General Brigham > MGH > Medical Services > General Internal Medicine
From:	Mass General Brigham IRB<br>399 Revolution Drive, Suite 710<br>Somerville, MA 02145
Title of Protocol:	PREdiction of ventilator Liberation Utilizing biomarker DiscovEry (PRELUDE)
Version/Number:	NA
Version Date:	
IRB Continuing Review/Amendment #:	5/36
IRB Review Type:	Expedited
Expedited Category/ies:	(5) Research involving materials (data, documents, records, or specimens) that have been collected, or will be collected solely for nonresearch purposes (such as medical treatment or diagnosis). (Note: Some research in this category may be exempt from the HHS regulations for the protection of human subjects. 45 CFR 46.101 (b)(4). This listing refers only to research that is not exempt.)
IRB Approval Date:	06/15/2022
Approval/Activation Date:	06/15/2022
**Next Review:**	Expedited Check In
**IRB Expiration Date:**	**06/15/2024**

This project has been reviewed and approved by the **Mass General Brigham IRB**. During the review of this project, the IRB specifically considered (i) the risks and anticipated benefits, if any, to subjects; (ii) the selection of subjects; (iii) the procedures for obtaining and documenting informed consent; (iv) the safety of subjects; and (v) the privacy of subjects and confidentiality of the data.

Please note that if an IRB member had a conflict of interest with regard to the review of this project, consistent with IRB policies and procedures, the member was required to recuse him/herself and, if

Mass General Brigham IRB
Mass General Brigham
399 Revolution Drive, Suite 710
Somerville, MA 02145
Tel: 857-282-1900
Fax: 857-282-5693

applicable, leave the room during the discussion and vote on this project except to provide information requested by the IRB.

As Principal Investigator, you are responsible for ensuring that this project is conducted in compliance with all applicable federal, state and local laws and regulations, institutional policies, and requirements of the IRB, which include, but are not limited to, the following:

1. Submission of any and all proposed changes to this project (e.g., protocol, recruitment materials, consent form, status of the study, etc.) to the IRB for review and approval prior to initiation of the change(s), except where necessary to eliminate apparent immediate hazards to the subject(s). Changes made to eliminate apparent immediate hazards to subjects must be reported to the IRB as an unanticipated problem.
2. Submission of a continuing review submission or institutional status report as required by the IRB and/or institution to continue the research, and submission of a final report when the project has been closed or completed.
3. Submission of any and all unanticipated problems, including adverse event(s) in accordance with the IRB's policy on reporting unanticipated problems including adverse events.
4. Obtaining informed consent from subjects or their legally authorized representative prior to initiation of research procedures when and as required by the IRB and, when applicable, documenting informed consent current IRB approved consent form(s) with the IRB-approval stamp in the document footer.
5. Informing all investigators and study staff listed on the project of changes and unanticipated problems, including adverse events, involving risks to subjects or others.
6. When investigator financial disclosure forms are required, submitting updated financial disclosure forms for yourself and for informing all site responsible investigators, co-investigators and any other members of the study staff identified by you as being responsible for the design, conduct, or reporting of this research study of their obligation to submit updated Investigator Financial Disclosure Forms for this protocol to the IRB if (a) they have acquired new financial interests related to the study and/or (b) any of their previously reported financial interests related to the study have changed.

IMPORTANT REMINDER: THE IRB HAS THE AUTHORITY TO TERMINATE PROJECTS THAT ARE NOT IN COMPLIANCE WITH THESE REQUIREMENTS.

Questions related to this project may be directed to IRB@partners.org

cc:

Jehan Alladina, Principal Investigator, General Internal Medicine, Medical Services

Kathryn Hibbert, MD, Co-Investigator, Pulmonary, Medical Services

Mamary Kone, MD, MPH, Research Coordinator/Manager, Pulmonary, Medical Services
