# Supplementary material for: Serial laboratory biomarkers are associated with ICU outcomes in patients hospitalized with COVID-19
Source: PLoS One. 2023 Nov 7;18(11):e0293842. doi: 10.1371/journal.pone.0293842 (PMC10629639; doi:10.1371/journal.pone.0293842)
Supplement: S2 Table — (DOCX) [file pone.0293842.s003.docx]

**Supplementary Table 2. Biomarker levels by COVID severity**
