# Supplementary material for: Serial laboratory biomarkers are associated with ICU outcomes in patients hospitalized with COVID-19
Source: PLoS One. 2023 Nov 7;18(11):e0293842. doi: 10.1371/journal.pone.0293842 (PMC10629639; doi:10.1371/journal.pone.0293842)
Supplement: S5 Table — (DOCX) [file pone.0293842.s006.docx]

**Supplementary Table 5. ROC analyses for 30-day, 60-day mortality, ICU transfer and OS**

Baseline model incorporates biomarker level on day 1 and baseline covariates; Baseline + serial change model included serial changes in each biomarker on top of the baseline model. Area under the curve (AUC) were calculated for 30-day, 60-day mortality, ICU transfer and C-index was calculated for OS. AUC was calculated based on 5-fold cross-validation and 95% confidence intervals (CIs) for AUC and C-index obtained by creating 300 bootstrap samples and taking the 2.5^th^ and 97.5^th^ percentiles of the re-estimates.
